# Lipidomics analysis facilitate insight into the molecular mechanisms of urate nephropathy in a gout model induced by combination of MSU crystals injection and high-fat diet feeding

**DOI:** 10.3389/fmolb.2023.1190683

**Published:** 2023-05-03

**Authors:** Guifeng Hao, Xiaofen Xu, Jingyi Song, Jida Zhang, Kejun Xu

**Affiliations:** ^1^ Center for General Practice Medicine, Department of Rheumatology and Immunology, Zhejiang Provincial People’s Hospital (Affiliated People’s Hospital, Hangzhou Medical College), Hangzhou, China; ^2^ College of Basic Medical Sciences, Zhejiang Chinese Medical University, Hangzhou, Zhejiang, China; ^3^ Emergency Medicine Department, The Second Affiliated Hospital of Zhejiang Chinese Medical University, Hangzhou, Zhejiang, China

**Keywords:** gout, hyperuricaemia, lipidomics, inflammation, cardiolipin, 4-hydroxyalkenal

## Abstract

Renal injury is one of the most common clinical manifestations of patients with hyperuricaemia/gout. The precise pathophysiological mechanism(s) for the renal injury is still unknown. Furthermore, it is also unclear whether the clinical therapies (e.g., colchicine and febuxostat) could prevent its progression or not. Lipids are involved in almost all of important biological processes and play critical roles in maintaining the renal functions. Herein, shotgun lipidomics was performed for class-targeted lipid analysis of cellular lipidomes in renal tissue of a gouty model induced by combination of monosodium urate crystals injection and high-fat diet feeding with/without treatment with either colchicine or febuxostat. Serum uric acid (UA), proinflammatory cytokines (i.e., TNF-α and IL-6), xanthine oxidase activity, footpad swelling, and pain threshold were determined to evaluate the gouty severity. Renal histopathological changes, blood urea nitrogen, creatinine, and kidney index were used to reflect renal injury. Lipidomics analysis revealed that altered triacylglycerol (TAG) profile, impaired mitochondrial function resulted by decreased tetra 18:2 cardiolipin, reduced 4-hydroxyalkenal (HNE) species, and elevated lysophospholipids were already present in the kidneys at early stage of renal injury, probably contributing to its occurrence and development. In addition to significantly reduce the UA level and relief the gouty severity, treatment with either colchicine or febuxostat could restore HNE bioavailability, thereby delaying the progression of renal injury. However, both of them could not recover the altered TAG profile and the impaired mitochondrial function, indicating that treatment with either of them could not completely prevent the development of renal injury in the gouty model.

## 1 Introduction

Gout is a chronic metabolic and inflammatory disease caused by deposition of monosodium urate (MSU) microcrystals in joints and soft tissues (Dalbeth, et al., 2016; Dalbeth, et al., 2021). So far, its pathogenesis could be mainly attributed to the formation of MSU crystals, which are resulted by elevated levels of serum uric acid (UA), in joints and these tissues surrounding the joints. In addition, the deposition of MSU further induce inflammatory responses with varieties of symptoms, including fever, swelling, redness, warmth, tenderness, pains, etc., (Feng, et al., 2022) Numerous studies have demonstrated that many risk factors could contribute to the development of gout, such as genetic, dietary, drugs, metabolic syndromes, and especially hyperuricaemia (Dalbeth, et al., 2016). Population-based studies from Asia, Europe, and North America display an increasing trend in the prevalence and incidence of hyperuricaemia and gout, already attracting more and more attention (Dalbeth, et al., 2016; Dalbeth, et al., 2021). Therefore, the central strategy for effective management of gout is long-term urate-lowing therapy to reverse hyperuricaemia, achieving the dissolution of MSU crystals and long-term prevention of gout flares. In clinical, febuxostat and colchicine are two kinds of representative agents for the effective treatment of gout through lowering the level of UA and inhibiting inflammatory responses, respectively (Abhishek, et al., 2017; Wen, et al., 2020). Nonetheless, treatment with these medicines could cause many side effects in gout patients, such as rash, diarrhea, vomiting, and particularly chronic renal toxicity (Wen, et al., 2020).

Accumulated studies have demonstrated that there exists close relationship between hyperuricaemia and kidney diseases (Xu, et al., 2017; Major, et al., 2018; Borghi, et al., 2020). Kidneys play a critical role in UA metabolism, for ∼65–75% of UA is excreted by renal tissues. The renal handling of UA includes glomerular filtration, tubular reabsorption, tubular secretion, and reabsorption after secretion. In theory, a reduced UA excretion and/or an elevated UA reabsorption inevitably result in hyperuricaemia, thereby contributing to the development of gout. Additionally, clinical studies have further proved this association. For instance, a 6-year cohort study including 10,677 Chinese individuals with a normal estimated glomerular filtration rat and without proteinuria indicated that a higher UA level contributed to the onset of kidney disease and a rapid decline of estimated glomerular filtration rat (Zhou, et al., 2019); a long-term follow-up cohort study showed a significant relationship between the baseline UA concentration and the risk of kidney disease (Weiner, et al., 2008); moreover, another cohort study lasted more than 25 years also revealed an independent association between high UA levels and end-stage renal diseases (Hsu, et al., 2009). Furthermore, many animal studies also demonstrated that hyperuricaemia is involved in the occurrence and development of various kidney diseases, including diabetic nephropathy, acute kidney injury, and chronic kidney disease (Wang, et al., 2016; Pan, et al., 2021; Wu, et al., 2021; Li, et al., 2022). So far, the pathophysiological mechanism(s) for hyperuricaemia-induced renal injury and their causal relationship in patients with gout remain unknown. Moreover, there are no sufficient evidences to clearly support that the usual therapies for gout in clinical, such as lowering the level of UA therapy (e.g., febuxostat) and inhibiting inflammatory responses therapy by treatment with colchicine, could effectively prevent the progression of renal injury.

Numerous studies have confirmed the relationship between inflammation and aberrant lipid metabolism (Hu et al., 2021b; Brennan, et al., 2021). It is well-known that inflammation plays a key role in pathogenesis of gout. In addition to serving as essential components of cell membranes, lipids and their related metabolites are also directly involved in many important biological processes, such as energy storage, signaling transduction, and cell growth, differentiation and survival (Han, 2016; Hu et al., 2022b). Moreover, accumulated studies also clearly demonstrated that the aberrant lipid metabolism could induce production of autoantibodies and increase levels of inflammatory cytokines, drastically accelerating the progression of lupus nephritis (Hu, et al., 2016; Hu et al., 2021a). Additionally, the lipid nephrotoxicity hypothesis that has been supported by more and more evidences indicates that decreased albumin levels, proteinuria, and the resultant hyperlipidemia could lead to a glomerulosclerosis analogous to atherosclerosis, further proving that lipid abnormalities contribute to the development of renal diseases as well as renal injury. Therefore, determination of lipid alteration could greatly facilitate understanding of the underling mechanism(s) responsible for the renal injury in patients with gout and revealing influences of different therapies for the progress of renal injury.

In the study, to comprehensively investigate the aberrant metabolism of lipids for renal injury, an advanced multi-dimensional mass spectrometry-based shotgun lipidomics (MDMS-SL) technology was performed for class-targeted lipid analysis of cellular lipidomes in renal tissue of a gout model induced by combination of MSU crystals injection and high-fat diet (HFD) feeding with/without treatment with medicines (i.e., febuxostat and colchicine). The analyzed lipids included triglycerides (TAGs), 4-hydroxyalkenal (HNE) species, various classes of phospholipids (e.g., cardiolipin (CL), choline glycerophospholipid (PC), ethanolamine glycerophospholipid (PE), phosphatidylserine (PS), and phosphatidic acid (PA)) and relevant lysophospholipids (such as choline lysoglycerophospholipid (LPC) and ethanolamine lysoglycerophospholipid (LPE)), sphingomyelin (SM) species, etc. In addition, renal histopathological evaluation, serum UA, blood urea nitrogen, proinflammatory cytokines measurement, and evaluation of footpad swelling and pain threshold of the mice from each group were also conducted for assessment of onset and/or outcome of gout.

## 2 Materials and methods

### 2.1 Materials

All synthetic phospholipids or other lipids, including 1,2-dimyristoleoyl-*sn*-glycero-3-phosphocholine (di14:1 PC), 1,2-dipalmitoleoyl-*sn*-glycero-3-phosphoethanolamine (di16:1 PE), 1’,3’-bis [1,2-dimyristoyl-snglycero-3-phospho]-glycerol (ammonium salt) (tetra 14:0 CL), 1,2-dimyristoyl-sn-glycero-3-phospho-L-serine (sodium salt) (14:0 PS), 1,2-dimyristoyl-sn-glycero-3-phosphate (sodium salt) (di14:0 PA), 1-heptadecanoyl-2-hydroxy-*sn*-glycero-3-phosphocholine (17:0 LPC), 1-myristoyl-2-hydroxy-sn-glycero-3-phosphocholine (14:0 LPE), triheptadecenoyl glycerol (T17:1 TAG), and 4-hydroxy-*9,9,9-d3*-2(*E*)-nonenal (4-HNE-*d3*) (100 μg in 200 μL of methyl acetate), used as internal standards were brought from Matreya, Inc. (Pleasant Gap, PA, United States), Avanti Polar Lipid, Inc. (Alabaster, AL, United States), or Cayman Chemical Co. (Ann Arbor, MI, United States). All the solvents and chemicals were at least the analytical grade and obtained from Sigma-Aldrich Chemical Company (St. Louis, MO, United States), Merck KGaA (Darmstadt, Germany), or Fisher Scientific (Pittsburgh, PA, United States).

### 2.2 Animal experiments

Specific pathogen-free (SPF) male C57BL/6 mice at 6 weeks of age were used in the study. The mice were obtained from Shanghai SLAC Laboratory Animal Co., Ltd. and housed in SPF environment of the Center Animal House of Zhejiang Chinese Medical University. All the procedures were conducted according to the Ethics Committee for the Use of Experimental Animals at Zhejiang Chinese Medical University (Approved No. IACUC-20220913–16). All information reported here were elaborated according to the ARRIVE guidelines. All mice were maintained in cages under a controlled environment with 12:12 light and dark cycle and an ambient temperature of 22–25°C. They had free access to food and water.

After 7 days of acclimatization, forty of C57BL/6 mice were randomly divided into four groups (ten mice per group): control group, gout model group, gout model-colchicine treatment group (the colchicine group), and gout model-febuxostat treatment group (the febuxostat group). The control group was fed with a standard diet and injected with 40 µL of PBS in the right hind footpad every 7 days. The other three groups were fed with HFD (10% yeast extract) and injected with MSU crystals (1 mg MSU crystals in 40 µL of PBS) every 7 days to establish gout model (Lin, et al., 2020). In addition, the colchicine and the febuxostat groups were administrated with colchicine (0.78 mg/kg/week) and febuxostat (5.2 mg/kg/day), respectively (Wen, et al., 2020). In addition, after the mice were injected with MSU crystals, the gout model-colchicine treatment group was treated with colchicine immediately, while the gout model-febuxostat treatment group was fed with febuxostat after 12 h to avoid the acute phase. The control and the model groups were orally fed with the same volume of distilled water.

After treatment for 6 weeks, mice were euthanized with CO_2_ and samples were collected at 12 h after the last administration and injection of MSU. Blood was collected by retro-orbital puncture, further centrifuged (1,300 *g*, 15 min, 4°C) to isolate serum samples. Renal tissue samples were removed for lipidomics analysis and histopathological evaluation. The tissue was lavaged with PBS until no blood was present within them, and stored at −80°C. Meanwhile, liver and foot joint tissues were also collected and then stored at −80°C.

### 2.3 Assessment of gouty onset and/or outcome

#### 2.3.1 Serum UA and proinflammatory cytokines measurement

An aliquot (200 µL) of serum samples of mice from different groups was used for measurement of serum uric acid and blood urea nitrogen. Their concentrations were determined by automatic biochemical analyzer (TOSHIBA TBA-120FR, Toshiba Medical Systems Co., Tochigi, Japan) according to manufacturer’s operation instructions. Proinflammatory cytokines (e.g., IL-1β and TNF-α) in serum samples of mice from different groups were measured by using the Mouse ELISA Commercial Kit (Jianglai Biotechnology Co., Ltd., Shanghai, China). The concentrations were calculated based on the standard curves.

In addition, XOD activity in hepatic tissue was also determined. Briefly, the mouse liver samples were pulverized into fine powder with a stainless-steel mortar and pestle at the temperature of liquid nitrogen. A powder sample from each liver tissue was further homogenized (50 mg per 1.0 buffer solution) and centrifuged (12,000 rpm, 10 min, 4°C) to obtain the supernatant. Then these supernatant samples were used for determination of XOD activity by following the manufacturer’s instructions (Jiangcheng, Nanjing, China).

#### 2.3.2 Evaluation of footpad swelling and pain threshold

According to the previous report, the footpad thickness of each mouse from different groups was measured with a caliper (Meinaite, Germany) before and at 4, 24, 48, and 72 h after injected MSU crystals into the footpads of the mice (Lin et al., 2020). The extent of foot swelling in mice was expressed as the ratio of Δmm/mm (at zero time point) of the joint. Von Frey filaments (Stoelting, Wood Dale, IL) were used to measure the mechanical retreat threshold to evaluate the foot pain threshold of mice as previously described (Chaplan et al., 1994).

### 2.4 Histopathological evaluation

Histopathological evaluation of footpad and renal tissue was conducted according to the previous report (Hu et al., 2015; Hu et al., 2021b). Mouse tissues were fixed in 4% paraformaldehyde and then subjected to routine paraffin embedding. Serial sections were cut at a thickness of 4–6 µm and stained with hematoxylin and eosin (H&E) and a modified Masson’s trichrome kit. All images were acquired by a Leica microscope equipped with a color CCD camera.

### 2.5 Preparation of lipid extracts from renal samples

As previously reported (Bligh and Dyer, 1959), the lipids of individual renal sample were extracted by using a modified protocol of Bligh and Dyer in the presence of internal standards (Han, 2005). Each lipid extract was resuspended with 2,000 µL chloroform/methanol (1:1, *v/v*)/mg protein, and stored at −20°C for lipid analysis. Derivatization of the primary amine in phosphoethanolamine-containing species (such as PE and lysoPE) with fluorenylmethoxycarbonyl chloride and HNE species with carnosine was performed according to the reported methods (Han, et al., 2005; Wang, et al., 2012; Sun, et al., 2022). Individual lipid species including FA isomers and regioisomers were identified through using multi-dimensional MS analysis (Yang, et al., 2009; Hu et al., 2022a). Incidentally, analysis of HNE species was finished with 1 week.

### 2.6 Lipid analysis, and data processing and analysis

According to the previous report (Han and Gross, 2005), a triple-quadrupole mass spectrometer (Thermo TSQ Quantiva, Thermo Fisher Scientific Inc., San Jose, CA, United States) equipped with an automated nanospray ion source (TriVersa NanoMate, Advion Bioscience Ltd., Ithace, NY, United States) was used for class-targeted lipid analysis of cellular lipidomes in renal samples. In order to prevent possible lipid aggregation, the solution of each lipid extract was diluted in CHCl_3_/MeOH/isopropyl alcohol (1:2:4, *v/v/v*) prior to direct infusion. The lipidomics analysis was conducted under a random experimental design with the experimental sequence determined by RAND function of Microsoft Excel (Office 2010; Microsoft Co., SFO, CA, United States). All mass spectral data were obtained by different customized sequence subroutines operated under Xcalibur software. Data processing was conducted as previously described (Yang, et al., 2009). The data were shown as mean ± SEM unless otherwise indicated. Statistical significance between the groups (*n* = 6) was determined using ANOVA followed by Dunn multiple comparison with IBM SPSS Statistics 19 Software (SPSS Inc., Chicago, IL, United States), where *p < 0.05, **p < 0.01, and ***p < 0.001.

## 3 Results

### 3.1 The gouty onset and severity, and/or outcome of mice from different groups

**FIGURE 1:**
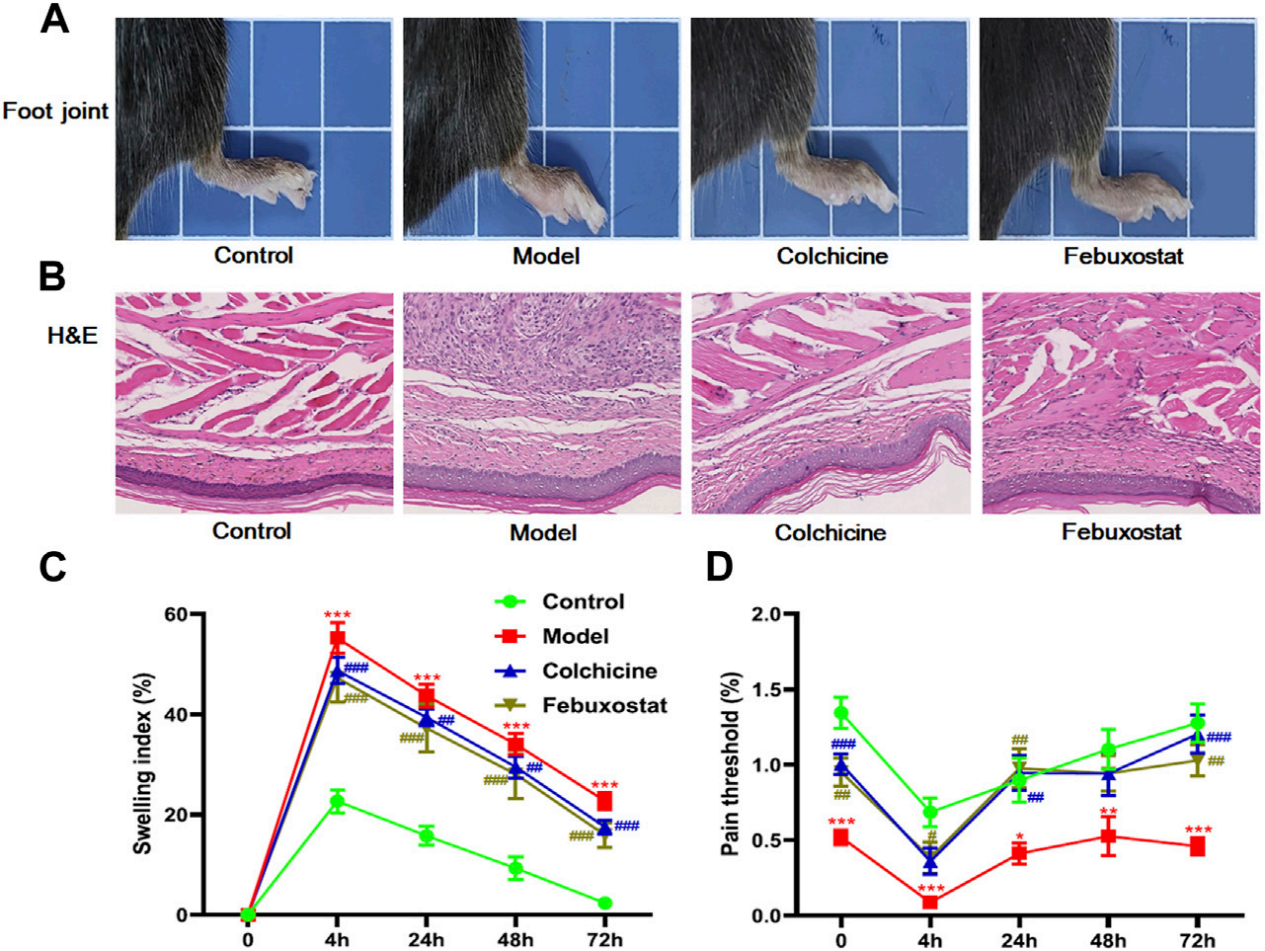
The gouty onset and severity, and/or outcome of mice from different groups. Representative images of foot joints **(A)** and hematoxylin and eosin **,** (H&E) staining analysis of the footpad **(B)**, footpad swelling index **(C)**, and footpad pain threshold **(D)** of mice from the control (*n = 10*), model (*n = 10*), colchicine (*n = 10*), and febuxostat (*n = 10*) groups. All representative images were captured under × 40 visual field **(B)**. The data present means ± SEM from different groups **(C, D)**. *p < 0.05, **p < 0.01, and ***p < 0.001 compared with those in the control group. ^#^ *p* < 0.05, ^##^ *p* < 0.01, and ^###^ *p* < 0.001 compared with those in the model group.

To evaluate the establishment of gout model and the efficiency of different treatments, the characteristic symptoms (e.g., the footpad swelling and the pain threshold) of mice from each group were assessed. In comparison with the control mice, the mice in model group displayed significant foot joint swelling and reduced footpad mechanical pain threshold after injected MSU (Figures 1A,C,D). Meanwhile, after treatment with either colchicine or febuxostat, foot joint swelling and footpad mechanical pain threshold of mice were greatly relieved, indicating these therapies could markedly alleviate these gouty symptoms (Figures 1A,C,D). Additionally, the images of H&E staining analysis of the footpad also clearly showed that MSU crystals significantly led to inflammatory cell infiltration and hyperplasia synovial in the foot joint in comparison with those of mice in control. Accordingly, these pathological alterations could also be attenuated to some extent through administrating with either colchicine or febuxostat (Figure 1B).

**FIGURE 2:**
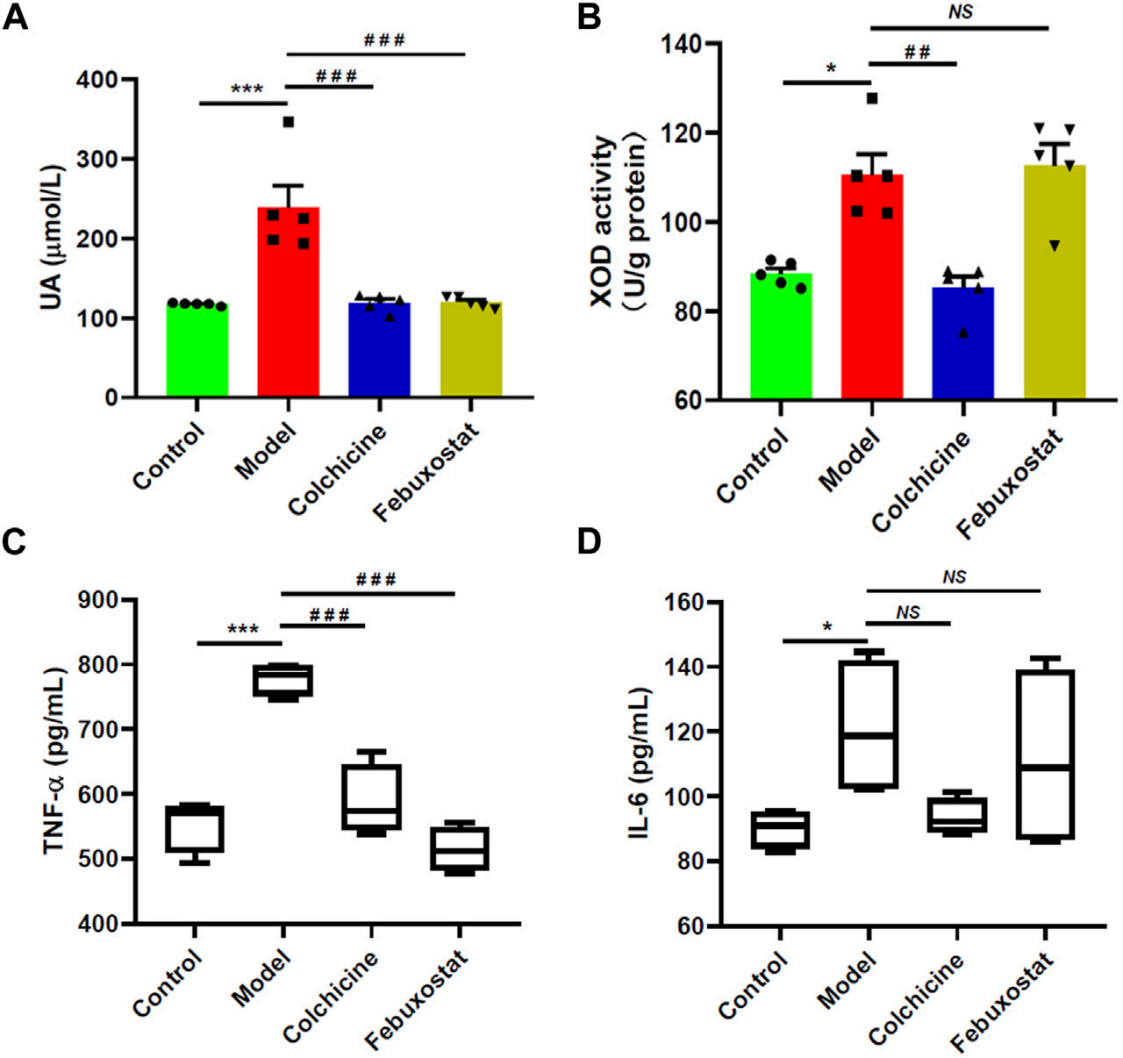
Comparison of levels of serum uric acid, xanthine oxidase activity, and proinflammatory cytokines of mice from different groups. The levels of serum uric acid (UA) **(A)**, xanthine oxidase (XOD) activity in the hepatic tissue **(B)**, the levels of TNF-α **(C)** and IL-6 **(D)** in serum samples of mice from the control (*n = 5*), model (*n = 5*), colchicine (*n = 5*), and febuxostat (*n = 5*) groups were determined with automatic biochemical analyzer and corresponding ELISA kits, respectively. The data present means ± SEM from different groups. *p < 0.05 and ***p < 0.001 compared with those in the control group. ^##^ *p* < 0.01 and ^###^ *p* < 0.001 compared with those in the model group. *NS*, not significant.

Moreover, the levels of serum UA and xanthine oxidase (XOD) activity in the hepatic tissue of mice from different groups were also measured with automatic biochemical analyzer and corresponding ELISA kit, respectively, for they are another important indexes of gout. The result clearly demonstrated that the concentration of serum UA showed similar alteration with the aforementioned indexes. Specifically, compared with that of the control mice, the serum UA in mice received injection of MSU crystals (the model group) drastically increased (*p* < 0.001, Figure 2A). Accompanying with substantially higher level of serum UA, the activity of enzyme XOD related to purine metabolism in the hepatic tissue of the model mice was elevated to some degree in comparison with that of the control mice (*p* < 0.05, Figure 2B). Accordingly, treatment with either colchicine or febuxostat could significantly reduce the level of serum UA (Figure 2A). As previously reported, some inflammatory cytokines, such as TNF-α and IL-6, were closely associated with inflammatory activity in patients with gout (Amaral, et al., 2016; Cavalcanti, et al., 2016). Thus, their levels in serum samples collected from mice of different groups were also determined with corresponding ELISA kits. As expected, the levels of both TNF-α and IL-6 were remarkably increased in the model group compared with those of the control mice (*p* < 0.001 and *p* < 0.05, respectively) (Figures 2C, D). After administrated with either colchicine or febuxostat, their concentrations were reduced to some degree, supporting that higher serum UA could result in inflammatory responses. Furthermore, the above results clearly suggested that either colchicine or febuxostat could effectively relieved gouty symptoms induced by combination of MSU crystals injection and HFD feeding.

### 3.2 Histopathological evaluation of renal tissues of mice from different groups

**FIGURE 3:**
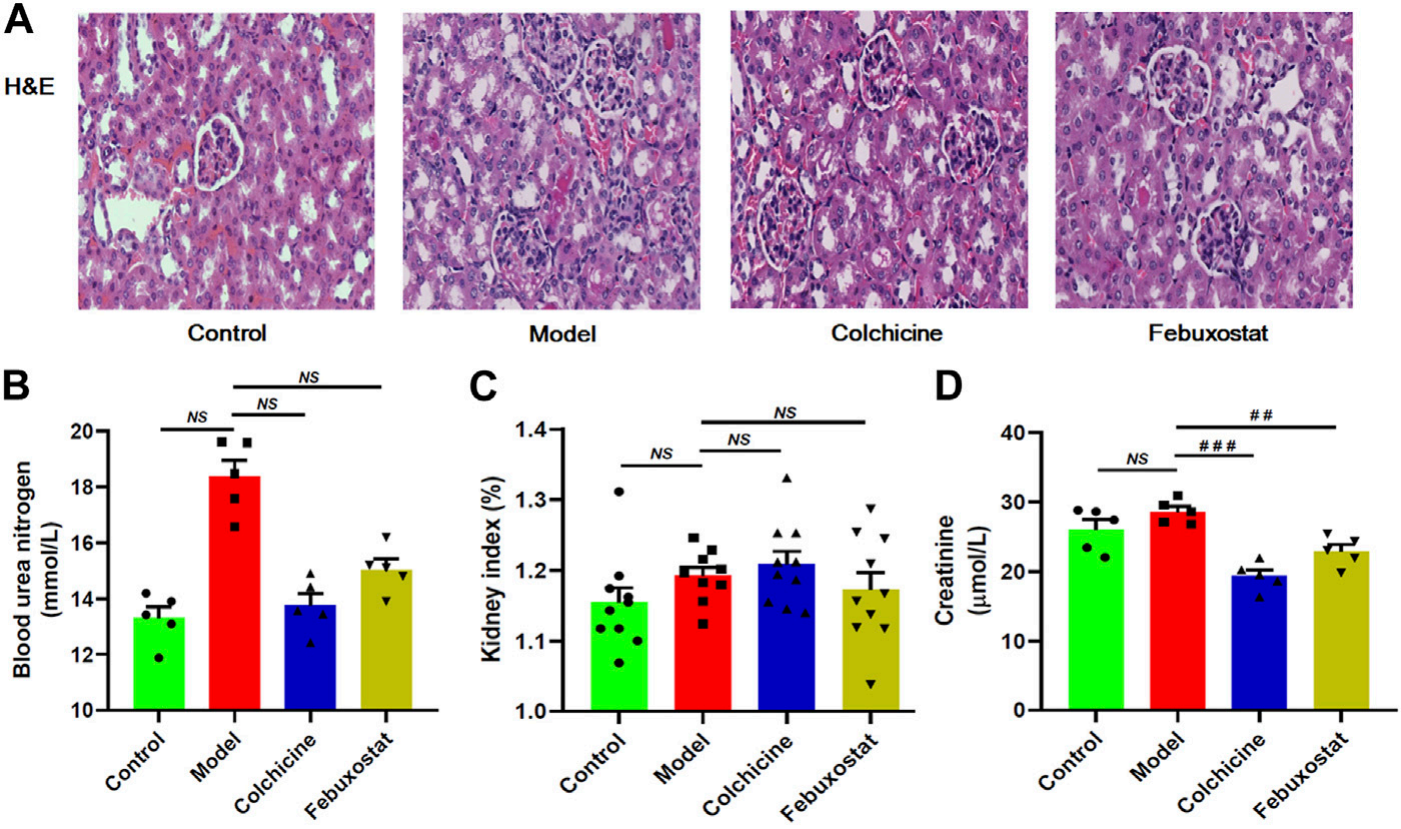
The onset and severity, and/or outcome of renal injury of mice from different groups. Representative images of hematoxylin and eosin **,** (H&E) staining analysis of renal tissue **(A)**, blood urea nitrogen **(B)**, kidney index **(C)**, and creatinine **(D)** of mice from the control, model, colchicine, and febuxostat groups. All representative images were captured under × 80 visual field **(A)**. The data present means ± SEM from different groups **(B–D)**. *NS*, not significant.

To assess the renal injury of mice, renal histopathological evaluation and blood urea nitrogen were also determined. The images of H&E staining clearly showed that some histopathological alterations (such as mesangial cell proliferation, glomerular swelling, and inflammatory cell infiltration) already existed in renal tissues of mice from the model. The representative images of H&E staining analysis of renal tissues were displayed in Figure 3A. Meanwhile, the level of blood urea nitrogen, which is one of the most important indicators of kidney function, also displayed increase trend in mice from the model group in comparison with the control group, although there was no significant difference between the two groups (Figure 3B). In addition, both kidney index (that is the ratio of kidney-to-mouse body weight) and level of creatinine of mice from the control and the model groups were similar (Figures 3C, D). These results collectively indicated that renal injury in the model mice was at early stage at this time point. Incidentally, treatment with either colchicine or febuxostat could correct these histopathological alterations to some extent, suggesting either of them could delay the development of renal injury (Figure 3A).

### 3.3 Lipidomics revealed altered composition of FA species in TAG pool in renal tissue of gout model mice

**FIGURE 4:**
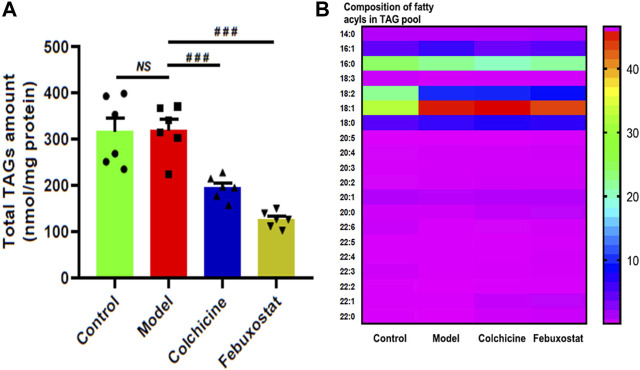
**C**omparison of the total mass level of triacylglycerol (TAG) species deposited in renal tissues of mice from different groups. Lipidomics analysis of total amount of TAG species **(A)** and composition of fatty acyls in TAG pool **(B)** present in lipid extracts of kidney from the control (*n = 6*), model (*n = 6*), colchicine (*n = 6*), and febuxostat (*n = 6*) groups was conducted by the multidimensional mass-spectrometry-based shotgun lipidomics technology. The data present means ± SEM from different groups **(A)**. ^###^ *p* < 0.001 compared with those in the model group. *NS*, not significant.

According to the lipid nephrotoxicity hypothesis, the resultant hyperlipidemia leads to a glomerulosclerosis similar to atherosclerosis with numerous lipids, especially TAGs, deposited in kidneys and/or other tissues, thereby contributing to development of renal injury as well as kidney disease (Hu, et al., 2017; Hu et al., 2021b). Moreover, in addition to hyperuricaemia, HFD also readily cause hyperlipidemia in logically. Hence, following this line of reasoning, the MDMS-SL technology was conducted to determine the levels of TAG species in the renal tissues of mice from different groups. The result of the lipidomics analysis demonstrated that there was no significant alteration of the total amount of TAGs in renal tissues of mice from the model group in comparison with the control (e.g., 315.97 ± 30.07 in controls and 319.70 ± 22.21 nmol/mg protein in models, respectively, *p* > 0.05) (Figure 4A). It indicated that the gout model induced by combination of MSU crystals injection and HFD feeding would not cause remarkable TAG deposition in renal tissue. In contrast, compared with those of the model group, the total amount of TAGs significantly decreased to 194.31 ± 10.34 and 125.48 ± 7.15 nmol/mg protein after treatment with either colchicine or febuxostat, respectively (*p* < 0.001, Figure 4A).

Although TAG deposition was not present in renal tissue of mice from the model group, it was found that, in comparison with those of the control, the composition of 18:2 FA in TAG pool of renal tissues substantially decreased (e.g., 22.81 ± 0.35 and 10.58 ± 0.20 mol% in controls and models, respectively, *p* < 0.001), whereas the composition of 18:1 FA markedly increased from 33.15 ± 0.48 in the control to 45.99 ± 0.65 mol% in the model (*p* < 0.001, Figure 4B). These predominant alterations collectively led to the significantly altered composition of FA species in TAG pool. Incidentally, these alterations could not be significantly corrected after treatment with either colchicine or febuxostat. Thus, the lipidomics analysis implied combination of MSU crystals injection and HFD feeding would induce the remarkably changed composition of 18:2 and 18:1 FAs in TAG pool of renal tissues, and either treatment with either colchicine or febuxostat could not correct these alterations.

### 3.4 Lipidomics demonstrated the aberrant metabolism of CL and HNE species in renal tissue of gouty mice

**FIGURE 5:**
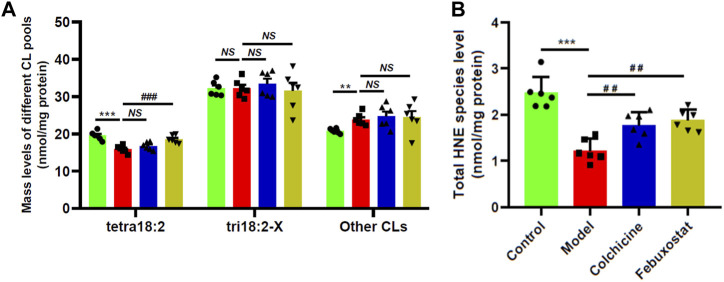
Comparison of the total mass levels of cardiolipin species and 4-hydroxyalkenal species in renal tissues of mice from different groups. Lipidomics analysis of total amount of different CL pools **(A)** and total HNE level **(B)** present in lipid extracts of renal tissues from the control (*n = 6, green*), model (*n = 6, red*), colchicine (*n = 6, blue*), and febuxostat (*n = 6, yellow*) groups was conducted by the multidimensional mass-spectrometry-based shotgun lipidomics technology. The data present means ± SEM from different groups. ***p < 0.001 compared with those in the control group. ^##^ *p* < 0.01 and ^###^ *p* < 0.001 compared with those in the model group. *NS*, not significant. CL and HNE denote cardiolipin and 4-hydroxyalkenal, respectively.

Thus, there existed one question that whether similar alterations in composition of 18:2 and 18:1 FAs were also present in other classes of lipids, such as CL. CL, which is a class of atypical phospholipids containing four fatty acyl chains and predominantly localized in the inner mitochondrial membrane, is very important for optimal mitochondrial functions (He and Han, 2014). CL, especially tetra18:2 CL, deficiency and/or improper remodeling of CL species could directly influence mitochondrial function. Therefore, the levels of CL species in renal tissue of mice from different groups were also determined by the MDMS-SL technology. It was found that in comparison with that of the control, tetra18:2 CL was significantly decreased in renal tissue of the model, leading to a decrease from 19.54 ± 0.48 in the control to 15.87 ± 0.40 nmol/mg protein in the model (*p* < 0.001, Figure 5A). Furthermore, the lower level tetra18:2 CL in the model could be attributed to the elevated level of improper remodeling of CL species that are difficult to remodel to tetra18:2 CL (e.g., 20.78 ± 0.26 and 23.86 ± 0.66 nmol/mg protein in controls and models, respectively) (*p* < 0.01, Figure 5A), while there existed no significant alteration in the total amount of tri18:2-X CLs that could be readily remodeled to tetra18:2 CL between the control and the model (Figure 5A). These results indicated that combination of MSU crystals injection and HFD feeding would induce the remarkably improper remodeling of CL species, thereby leading to impaired mitochondrial function in renal tissues of the model. It should be noted that treatment with either colchicine or febuxostat could not significantly relieve most of those alterations in different CL pools (Figure 5A).

It is well-known that CL species are functionally involved in mitochondrial bioenergetics, including optimal activities of respiratory chain complexes and ADP-ATP translocase, cytochrome c anchoring to the out leaflet of the inner mitochondrial membrane, regulating mitochondrial dynamics, etc (Oemer, et al., 2021). CL species are also potent reactive oxygen species (ROS) scavengers due to their high content in polyunsaturated FAs and location near the site of ROS production (Shi, 2010). Therefore, the reduced amount of tetra18:2 CL in renal tissue of the model could lead to increased oxidative stress to some extent. HNE species are the end products of lipid peroxidation of polyunsaturated fatty acids, serving as an indicator of lipid peroxidation and the degree of oxidative stress (Hu, et al., 2016; Hu et al., 2021a). Thus, the MDMS-SL technology was performed to determine the levels of HNE species in renal tissue of mice from different groups. Intriguingly, lipidomics analysis revealed that the total amount of HNE species was drastically decreased from 2.47 ± 0.14 in the control to 1.22 ± 0.10 nmol/mg protein in the model (∼50 mol% reduction, *p* < 0.001) (Figure 5B). Depending on the previous report, UA serving as a major antioxidant could effectively remove superoxide and ROS in human body (Su, et al., 2020). Thus, the reduced amount of HNE species in the model group might be due to higher level of UA. Furthermore, in parallel with the decreased UA, the total level of HNE species in either colchicine or febuxostat group was significantly increased in comparison with that of the model, respectively (Figure 5B). These results further demonstrated that higher concentration of UA could inhibit the production of HNE species.

### 3.5 The alterations of major phospholipids and lysophospholipid species in renal tissue of mice from different groups

**FIGURE 6:**
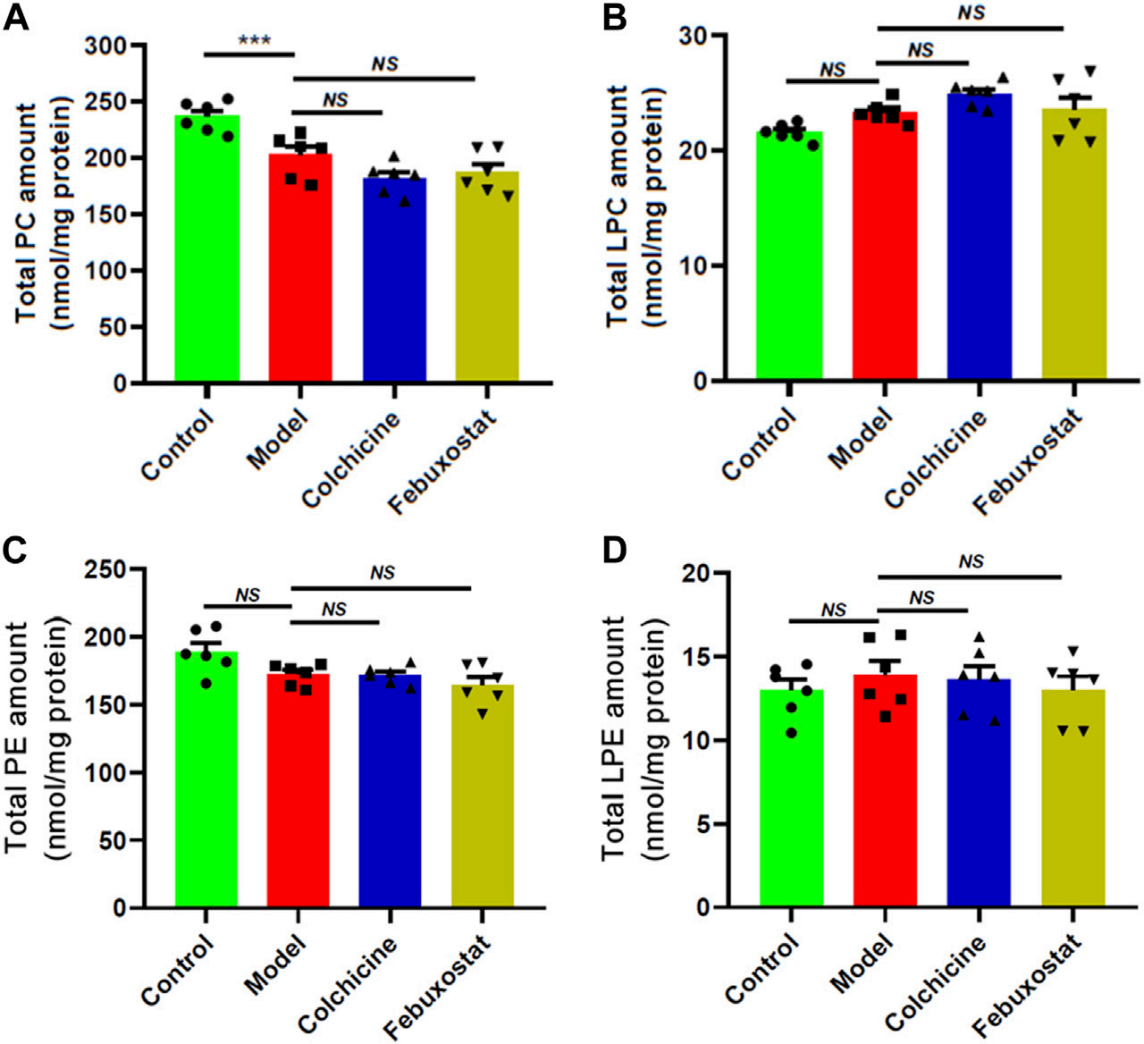
Comparison of the total mass levels of major phospholipids and relevant lysophospholipid species in renal tissues of mice from different groups. Lipidomics analysis of total amounts of choline glycerophospholipid (PC) **(A)**, choline lysoglycerophospholipid (LPC) **(B)**, ethanolamine glycerophospholipid (PE) **(C)**, and ethanolamine glycerophospholipid (LPE) species **(D)** present in lipid extracts of kidney from the control (*n = 6*), model (*n = 6*), colchicine (*n = 6*), and febuxostat (*n = 6*) groups was performed by the multidimensional mass-spectrometry-based shotgun lipidomics technology. The data present means ± SEM from different groups. ***p < 0.001 compared with those in the control group. *NS*, not significant.

Lower concentration of HNE could activate the NF-κB pathway, thereby leading to increased proinflammatory cytokine expression (Sharma, et al., 2022). In general, elevated concentrations of lysophospholipids as well as lower levels of corresponding phospholipid species exist in certain inflammatory states, for their play important roles in inflammation (Knuplez and Marsche, 2020). Therefore, two major phospholipids (i.e., PC and PE) and their relevant lysophospholipid species (i.e., LPC and LPE) present in the renal tissues from different were determined by the MDMS-SL technology. The lipidomics analysis suggested that compared with that of the control, the total level of LPC species in the model displayed an upward tendency, whereas the total amount of PCs in the model showed declining trend, although there existed no marked differences between the two groups (*p* > 0.05, Figures 6A,B). Moreover, the similar change trends were also present in the alterations of PE and LPE species (Figures 6C,D). These results could be attributed to the fact that the severity of inflammation in renal tissue of the model mice was not serious at this time point. Furthermore, treatment with either colchicine or febuxostat did not change these alteration trends (Figure 6).

### 3.6 The alterations of other classes of phospholipids and sphingomyelin species in renal tissue of mice from different groups

**FIGURE 7:**
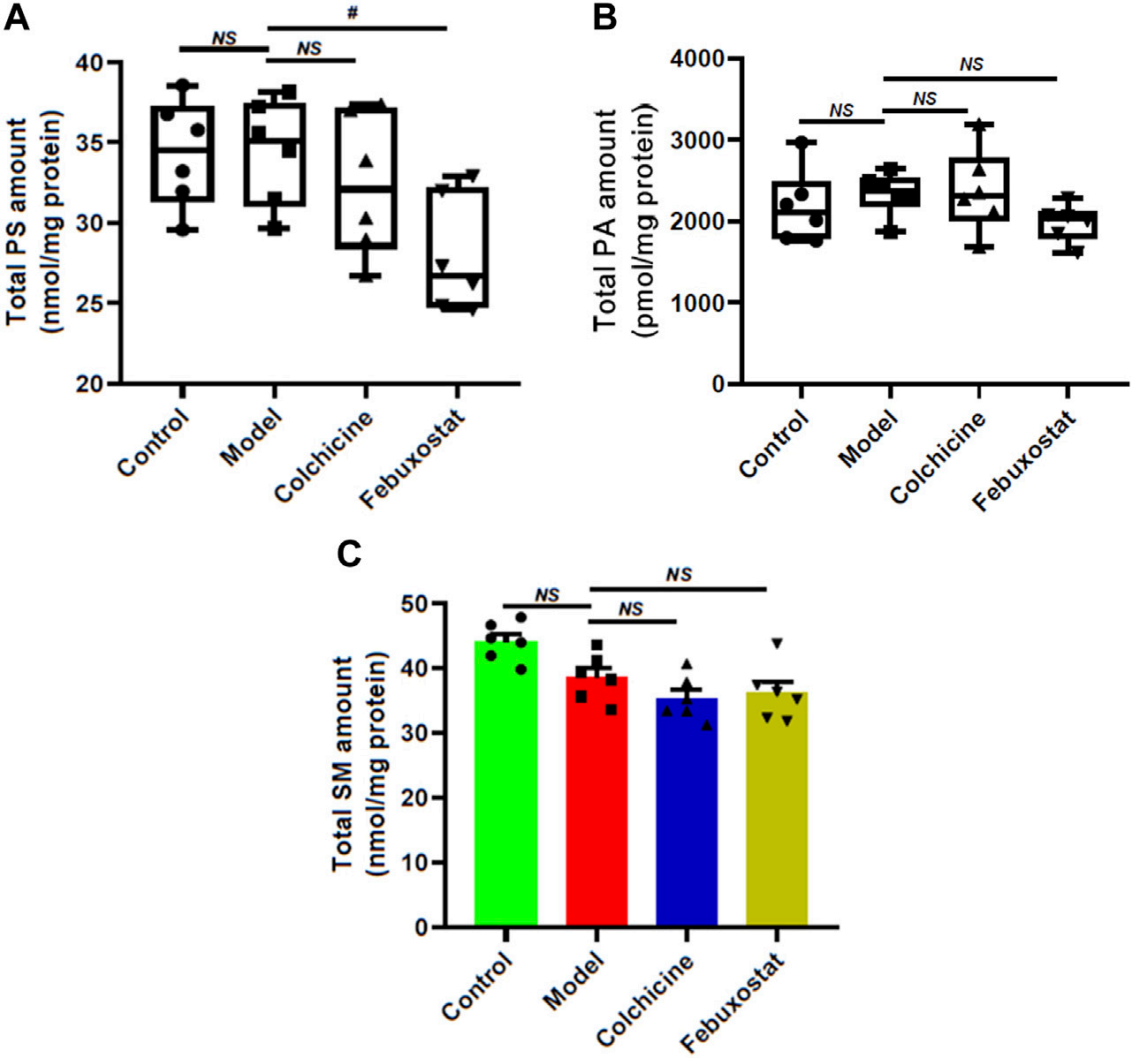
Comparison of the total mass levels of other classes of phospholipids and sphingomyelin species in renal tissues of mice from different groups. Lipidomics analysis of total amounts of PS **(A)**, PA **(B)**, and SM species **(C)** present in lipid extracts of kidney from the control (*n = 6*), model (*n = 6*), colchicine (*n = 6*), and febuxostat (*n = 6*) groups was conducted by the multidimensional mass-spectrometry-based shotgun lipidomics technology. The data present means ± SEM from different groups. ^#^ *p* < 0.05 compared with those in the model group. *NS*, not significant.

In order to further reveal aberrant lipid metabolism, the MDMS-SL technology was used to determine the other classes of phospholipids (e.g., PS and PA) and SM species. Figure 7 summarized the alterations of these lipid classes in renal tissues of mice from each group. Briefly, compared with these of the control, PS (control vs. model: 34.37 ± 1.36 vs. 34.45 ± 1.34 nmol/mg protein, *p* > 0.05) and PA (2178.68 ± 181.90 in the control to 2338.16 ± 107.56 pmol/mg protein, *p* > 0.05) did not show remarkable changes, while SM displayed declining tendency in the model (control vs. model: 44.10 ± 1.21 vs. 38.56 ± 1.50 nmol/mg protein, *p* > 0.05) (Figure 7). Additionally, after treatment with either colchicine or febuxostat, the total amounts of PS, PA and SM in renal tissues did not show significant alterations to some degree (Figure 7).

## 4 Discussion

Renal disease as well as renal injury, including chronic urate nephropathy, acute urate nephropathy, and urate nephrolithiasis, is one the most common manifestations in the patients with hyperuricaemia/gout (Li, et al., 2022). Lipids play key roles in the functions of kidney. In the present study, to reveal the mechanism(s) for the renal disease and evaluate the impacts of different therapies for its progression, the MDMS-SL technology was used for class-targeted lipid analysis of cellular lipidomes in renal tissue of a gout model induced by combination of MSU crystal injection and HFD feeding with/without treated with either febuxostat or colchicine.

Hyperuricaemia induced significantly changed composition of FAs, especially 18:2 and 18:1 FAs, in TAG pool of renal tissues, probably serving an important role in the occurrence of renal disease. Although the renal histopathological evaluation, the level of blood urea nitrogen, and the kidney index demonstrated that the functions of kidney from the model mice had not been seriously damaged at this time point, lipidomics analysis clearly revealed that aberrant lipid metabolism was already present in renal tissues (Figure 3). Based on the previously reported, higher UA could induce TAG accumulation that ectopically deposit in kidney, thereby contributing to the renal damage through toxic processes named lipotoxicity (Lanaspa, et al., 2012; Izquierdo-Lahuerta, et al., 2016). The result of lipidomics analysis unambiguously suggested that no remarkably ectopic fat deposition happened in the renal tissue at early stage of renal disease (Figure 4A). Although the amount of TAGs in renal tissue from the model mice was not significantly increased, the FA profiles of TAGs were altered markedly, particularly 18:2 and 18:1 FAs (Figure 4B), being consistent with the previous reports on alteration in TAG profile in patients with advancing chronic kidney disease (Afshinnia, et al., 2018). As we know, the functional properties of TAGs also depend on its FA profiles. On the one hand, alteration of the molecular structure of TAG leads to changes in melting/dropping point, crystallization behavior, thermal properties, and oxidative stability, consequently resulting in alterations in the glomerular filtration barrier and renal functions. On the other hand, studies have also demonstrated that monounsaturated FA, such as 18:1 FA, could protect cultured proximal tubules and other cell cultures from palmitate lipotoxicity (Ackerman, et al., 2018). Furthermore, TAG “loading” with more 18:1 before cellular injury could protect against cytotoxic stress that results in accumulation of saturated FA (Baek, et al., 2022). Following the reasoning line, the alteration in TAG profile in renal tissues from the model mice might serve as an adaptive response to prevent renal disease. Therefore, further studies are required to fully understand how the TAG profiles affect the development of renal disease induced by hyperuricaemia.

Impaired mitochondrial function induced by UA contributed to the progress of renal disease. The kidney is a highly energy-demanding organ that relies heavily on β-oxidation of FA for fuel. Thus, renal tissue has a large mitochondrial content to generate lots of ATP. In addition to the significant alteration of TAG profile in renal tissue of the model mice, lipidomics analysis clearly indicated that the level of improper remodeling of CL species was substantially elevated, consequently leading to the lower amount of tetra18:2 CL in the model mice (Figure 5A). It is well-known that tetra18:2 CL serves as an important index of mitochondrial function. Its reduced level reflected mitochondrial dysfunction and thereby impaired β-oxidation of FA in renal tissue of the model mice. This result was consistent with previous reports on mitochondrial function in kidney disease (Afshinnia, et al., 2018; Baek, et al., 2022). It has been demonstrated that reduced β-oxidation of FA and resultant insufficient ATP production is a major mechanism of tubular injury and fibrosis (Kang, et al., 2015). Moreover, restoring mitochondrial morphology and dysregulation of β-oxidation of FA could inhibit the development of kidney disease, further supporting that downregulation in β-oxidation of FA caused by reduced tetra18:2 CL is a key driver of kidney disease (Miguel, et al., 2021). Therefore, decreased tetra18:2 CL resulted by higher improper remodeling of CL species in renal tissue from the model mice impaired mitochondrial function and β-oxidation of FA, contributing to the development of renal disease.

The aberrant lipid metabolism caused by higher serum UA also could further promote inflammation responses, accelerating the process of renal injury. On the one hand, it is generally accepted that hyperuricaemia directly cause kidney injury by a crystal-dependent mechanism. For instance, MSU crystals deposited in the tubular lumen or interstitial space in the kidney can be recognized and engulfed by macrophages that reside or infiltrate the renal system, leading to chronic inflammation and substantial tubular damage (Liu, et al., 2015; Su, et al., 2020; Wu, et al., 2021). Moreover, MSU crystals could induce chemokines, such as CXCL-12, which induce directional proinflammatory cytokines, including IL-1β, IL-18, and interferons (Välimäki, et al., 2013). On the other hand, the aberrant metabolism of lipids induced by hyperuricaemia also stimulated inflammation responses and caused the destruction of renal tissue, further promoting the progression of renal disease. Lipidomics analysis demonstrated that the antioxidant activity of UA led to the lower level of HNE species in renal tissue from the model mice, reducing the renal HNE bioavailability (Figure 5B). Although HNE species are still considered to a toxic end product of lipid peroxidation, accumulated studies have demonstrated that HNE species can be beneficial or detrimental to cells/tissues depending on the concentration (Sharma, et al., 2022). Lower levels of HNE species lead to the induction of transcription factor Nrf2 and NF-κB, thereby resulting in the release of proinflammatory cytokines, including IL-1β, IL-8, and TNF-α. In addition to destroy the integrity of membrane structure and cause cell lysis for their amphipathic characteristics, the elevated lysophospholipids (e.g., LPC and LPE) present in renal tissue from the model mice could promote the expression of adhesion molecules and induce monocyte chemotaxis and pro-inflammatory cytokine production from macrophages (Tan, et al., 2020). Therefore, the aberrant lipid metabolism caused by higher UA could aggravate the development of renal disease in gouty model.

Additionally, treatment with either colchicine or febuxostat could partially correct the aberrant lipid metabolisms in gouty model, thereby preventing the progression of renal disease to some extent. In clinical, febuxostat and colchicine are two kinds of representative agents for the management of gout, although their mechanisms on the treatment for gout are different. In the study, the images of HE staining analysis of renal tissue showed that either colchicine or febuxostat could prevent the development of renal injury in the gouty model to some degree (Figure 3A). It might be attributed to the facts that all of them could effectively reduce the level of UA, inhibiting the resultant inflammatory responses (Figures 2A,C) and increasing the renal HNE bioavailability (Figure 5B). However, treatment with either colchicine or febuxostat could not completely correct the altered TAG profile and decrease the level of improper remodeling CL species to restore mitochondrial homeostasis (Figure 4B; Figure 5A). Thus, treatment with either colchicine or febuxostat could delay but not totally prevent the development of renal injury in the gouty model.

In summary, the present study clearly demonstrated that the aberrant metabolism of lipids was already present in the early stage of renal injury in the gout model induced by combination of MSU crystals injection and high-fat diet feeding. In addition to chronic inflammation and renal damage directly caused by deposition of MSU crystals, lipidomics analysis revealed that altered TAG profile, impaired mitochondrial function, and inflammation responses induced by reduced HNE species and elevated lysophospholipids also could contribute to the development of renal injury. In addition, treatment with either colchicine or febuxostat could effectively reduce the UA level and restore HNE bioavailability, but did not recover the altered TAG profile and the impaired mitochondrial function. It indicated that treatment with either colchicine or febuxostat could delay but not totally prevent the development of renal injury in the gouty model.

## Data Availability

The original contributions presented in the study are included in the article/Supplementary Materials, further inquiries can be directed to the corresponding author.

## Abbreviations

CL, cardiolipin; HFD, high-fat diet; 4-HNE, 4-hydroxyalkenals; MSU, monosodium urate; MDMS-SL, multi-dimensional mass spectrometry-based shotgun lipidomics; lysoPC, choline lysoglycerophospholipid; lysoPE, ethanolamine lysoglycerophospholipid; MDMS-SL, multi-dimensional mass spectrometry-based shotgun lipidomics; PA, phosphatidic acid; PE, ethanolamine glycerophospholipid; PC, choline glycerophospholipid; PS, phosphatidylserine; SM, sphingomyelin; TAG, triacylglycerol; UA, uric acid; XOD, xanthine oxidase.
